# Biomechanics and clinical outcome after posterior stabilization of mid-thoracic vertebral body fractures: a systematic literature review

**DOI:** 10.1007/s00068-020-01560-5

**Published:** 2020-12-02

**Authors:** Ulrich J. Spiegl, Georg Osterhoff, Philipp Bula, Frank Hartmann, Max J. Scheyerer, Klaus J. Schnake, Bernhard W. Ullrich

**Affiliations:** 1 Department of Orthopaedics, Trauma Surgery and Plastic Surgery, University of Leipzig, Liebigstr. 20, 04103 Leipzig, Germany; 2 Department of Orthopaedics and Trauma Surgery, Klinikum Gütersloh, Gütersloh, Germany; 3 Center for Trauma and Orthopedic Surgery, Gemeinschaftsklinikum Mittelrhein, Ev. Stift, Koblenz, Germany; 4 Department of Orthopedics and Trauma Surgery, University Hospital of Cologne, Cologne, Germany; 5 Center for Spinal and Scoliosis Surgery, Malteser Waldkrankenhaus St. Marien, Erlangen, Germany; 6 Department of Trauma Surgery and Reconstructive Surgery, BG Klinikum Bergmannstrost, Halle, Germany

**Keywords:** Thoracic spine fractures, Posterior stabilization, Clinical outcome, Pedicle screw placement, Additional thoracic injuries

## Abstract

**Purpose:**

The aim of this review is to systematically screen the literature for clinical and biomechanical studies dealing with posterior stabilization of acute traumatic mid-thoracic vertebral fractures in patients with normal bone quality.

**Methods:**

This review is based on articles retrieved by a systematic search in the PubMed and Web of Science database for publications up to December 2018 dealing with the posterior stabilization of fractures of the mid-thoracic spine.

**Results:**

Altogether, 1012 articles were retrieved from the literature search. A total of 960 articles were excluded. A total of 16 articles were dealing with the timing of surgery in polytraumatized patients, patients suffering of neurologic deficits after midthoracic fractures, and the impact of concomitant thoracic injuries and were excluded. Thus, 36 remaining original articles were included in this systematic review depicting the topics biomechanics, screw insertion, and outcome after posterior stabilization. The overall level of evidence of the vast majority of studies is low.

**Conclusion:**

High quality studies are lacking. Long-segmental stabilization is indicated in unstable midthoracic fractures with concomitant sternal fractures. Generally, long-segmental constructs seem to be the safer treatment strategy considering the relative high penetration rate of pedicle screws in this region. Thereby, navigated insertion techniques and intraoperative 3D-imaging help to improve pedicle screw placement accuracy.

## Introduction

The majority of traumatic vertebral fractures occur at the thoracolumbar junction and less commonly at the mid-thoracic or mid-lumbar spine [32]. In accordance, a high number of articles are dealing with thoracolumbar fractures focusing on the thoracolumbar junction. Nonetheless, the anatomy and biomechanics of the mid-thoracic spine differ from the thoracolumbar junction. First of all, the vertebral bodies including the pedicles are smaller at the thoracic spine, the orientation of the facet joints is different allowing rotational motion [44]. Next, the thoracic cage, defined as the fourth column by several authors, stabilizes the thoracic spine and leads to a higher stiffness [39]. Last but not least, the sagittal alignment of the thoracic spine consisting of a kyphosis differs tremendously from the thoracolumbar junction and the lordotic lumbar spine [33].

Based on these differences, the thoracic spine needs to be analyzed separately. According to literature, posterior stabilization is the most common used treatment strategy in unstable mid-thoracic fractures [37].

The aim of this review is to systematically review the literature for clinical and biomechanical studies dealing with posterior stabilization of acute traumatic mid-thoracic vertebral fractures. From this, the current state of evidence considering all aspects of the posterior stabilization shall be described. Based on these results, prospective studies could be created to increase the evidence in this field.

## Methods

The literature search included unstable recent vertebral fractures (< 4 weeks) of the mid-thoracic spine (Th 2—Th 10) of adults treated by posterior stabilization with adequate trauma history. Children and adolescents (age < 18) and elderly (age > 65) with likely concomitant osteopenia/osteoporosis were not within the scope of this review and need to be analyzed separately. Furthermore, patients with fractures after non-adequate trauma (trivial falls from tripping) were not included in this review.

A systematic search of the literature was performed by two of the authors (UJS, BWU), including all articles until 12/26/2018. In each case, the two databases PubMed and Web of Science Core Collection were considered and searched. Excluded were articles dealing with osteoporotic or pathologic vertebral body fractures, cervical and/or lumbar vertebral body fractures, and exclusively non-operative therapy strategies. Furthermore studies dealing with the timing of surgery in polytraumatized patients, patients suffering of neurologic deficits after midthoracic fractures, and the impact of concomitant thoracic injuries and were excluded. Additionally, case reports, reviews, and animals studies were excluded. Since data collection had already been completed at the time of PROSPERO registration, this review could not be registered with PROSPERO. Using the PICO scheme [11], the following review questions were defined:

- What is the recommended insertion technique of pedicle screws at the mid-thoracic spine?
- Short or long segmental stabilization—what should be preferred
- What is the expected outcome of fractures of the mid-thoracic spine treated by posterior stabilization?

The following search terms were used: “thoracic vertebral body fractures” OR “thoracic vertebral spine fractures” NOT “Osteoporosis” NOT “case report” NOT “tumor” NOT “lumbar spine”.

Subsequently, all relevant original articles were analyzed based on their levels of evidence and their appropriate conclusions. Here, the following topic areas were defined:

- Biomechanics
- Screw insertion
- Outcome after posterior stabilization

## Results

**Fig. 1:**
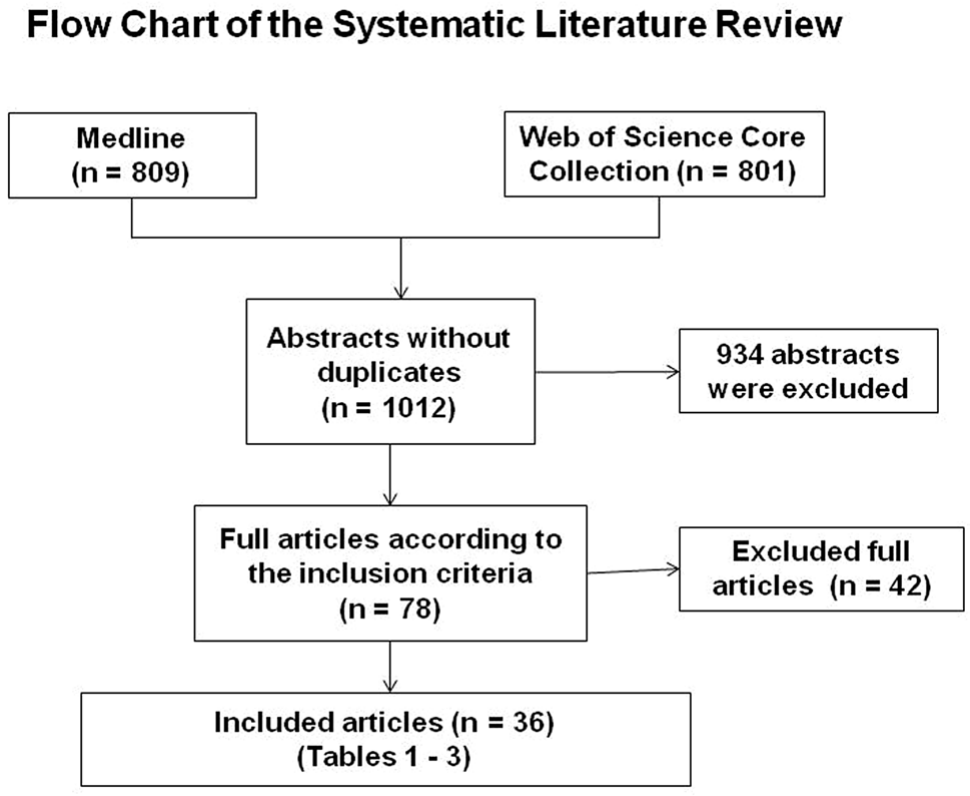
Flow chart of the systematic literature review

Altogether, 1012 abstracts were retrieved from the literature search (Fig. 1). Of these, articles were excluded based on abstract or title. Most of the excluded studies were overlaps between both databases, animals studies, no original articles or were articles investigated other pathologies or included cervical or lumbar factures, or exclusively evaluated non-operative treatment or anterior approaches. Altogether, 78 articles were analyzed completely. Of these articles 26 were additionally excluded, not focusing specifically on the thoracic spine, including geriatric patients or insufficiently describing the method of posterior stabilization. A total of 16 articles analyzed the timing of surgery in polytraumatized patients, reported of patients suffering of neurologic deficits after midthoracic fractures, and evaluated the impact of concomitant thoracic injuries and were excluded. Altogether, 976 articles were excluded (Fig. 1). All 36 remaining original articles, which covered the period from 1971 to 2018 are summarized in Tables 1, 2 and 3. Levels of evidence were defined as described by Bassler and Antes [1].

**Table 1:** Biomechanical studies

Study	Purpose	Key message
Watkin et al. [43] (2005)	Amount of stability provided by the rib cage	The intact rib cage provides high stability in all motion directions Sternal fractures decrease stability particularly in extension-flexion motion
Perry et al. [30] (2014)	Long segment versus short segment instrumentation in thoracic burst fracture	Long segment instrumentation superior during flexion–extension During lateral bending and axial rotation short and long instrumentations are comparable
Lazaro et al. [24] (2011)	Long segment instrumentation versus short instrumentation with cross-links and/or index screws	Index screws increase stability about 25% Cross-links only stabilize during axial rotation
Hongo et al. [18] (2009)	Metal clamps combined with polyester belts versus sublaminar wiring versus hooks versus pedicle screws	Pedicle screw fixation is superior
Little et al. [26] (2010)	Costotransverse process screws versus pedicle screws	Pedicle screw fixation is superior

**Table 2:** Studies dealing with pedicle screw placement

Study (year)	Purpose	Study design	No of screws	Main message	Ev-L
Vaccari et al. [40] (1995)	Pedicle screw insertion in Roy-Camille technique	Cadaver study	90	Screw penetration rate of 41%	n.a
Kothe et al. [22] (2001)	Navigated parapedicular insertion technique	Cadaver study	54	Safe and reliable technique	n.a
Reidy et al. [31] (2001)	Is intraoperative EMG-monitoring necessary	Prospective cohort study	95	No improvement in accuracy could be observed	II
Husted et al. [21] (2003)	Parapedicular approach	Cadaver study	24	No spinal canal penetration No pleural or foraminal penetration	n.a
Schnake et al. [35] (2004)	Navigated screw insertion	Prospective cohort study	324	Significant reduction in all screw penetrations Significant reduction in relevant screw penetration	II
Mac-Thiong et al. [27] (2003)	Evaluation of special drill guide for pedicle screw accuracy	Cadaver study	66	No screw penetration of more than 2 mm could be seen	IV
Bransford et al. [4] (2006)	Accuracy of free-hand pedicle screw placement under fluoroscopic control	Retrospective case series	1533	No major complication was observed 1.2% of prophylactic revision surg. due to screw malposition	IV
Dhawan et al. [9] (2008)	Comparison of the pedicle screw insertion direction	Cadaver study	966	The anatomic position leads to a 20% larger effective pedicle diameter	n.a
Chan et al. [6] (2010)	Evaluating the funnel technique	Cadaver study	240	No medial pedicle penetration by direct visualization via wide laminectomy	n.a
Wu et al. [43] (2010)	Free-hand versus navigated pedicle screw placement	Prospective randomized trial	176	Significant less pedicle screw penetrations with navigation Significant reduced radiation time with navigation Faster pedicle screw placement with navigation	II
Beck et al. [2] (2012)	Benefit of intraoperative 3D-imaging for pedicle screw accuracy	Prospective case series	240	3.8% intraoperative pedicle screw revision 2.5% of postoperative pedicle screw penetration, all grade I (< 2 mm)	III
Lehmann et al. [25] (2014)	Identification of the ideal starting point of pedicle screw insertion	Cadaver study	229	2–3 mm lateral to the midline of the superior articular facet	n.a
Cho et al. [7] (2015)	Feasibility of translaminar screws	Cadaver study	294	Safe technique Small number of moderate penetrations	n.a
Gonzalvo et al. [16] (2015)	Pedicle penetration rate in dependeny of the pedicle diameter	Retrospective case series	218	Penetration rate of 33%: pedicles diameter < 5 mm Penetration rate of 11%: Pedicle diameter 5–7 mm No penetrations if pedicle diameter > 7 mm	IV
Hu et al. [19] (2015)	Anatomic feasibility of translaminar screw placement	Cadaver study	6	Translaminar screws are not limited by anatomy in Asian patients	n.a
Kwan et al. [23] (2015)	Comparison of pedicle screw accuracy between percutaneous and open placement	Cadaver study	288	Percutaneous technique has a similar accuracy compared to the open placement Penetration rate: 11% open versus 8% percutaneous	n.a
Fischer et al. [12] (2016)	Gguide wire-based pedicle screw insertion under CT-imaging	Retrospective case series	286	Very high accuracy and low complication rate using this technique	IV

**Table 3:** Clinical outcome studies

Study (year)	Purpose	Study design	N	FU (months)	Main message	Ev-L
Capen et al. [5] (1994)	Non-operative treatment of upper thoracic spine fractures	Retrospective case series	49	No data	Conservative treatment of fractures from T1 to T8 can be successful	IV
Harkönen et al. [17] (1979)	Non-operative treatment of thoracic spine fractures	Retrospective case series	98 (75)	64	Non-operative treatment did not prevent aggravation of rad.deformity Clinical results were poor in patients with deformities	IV
Zifko et al. [48] (1971)	Non-operative treatment of thoracic spine fractures	Retrospective case series	271 (132)	60–108	Fractures with involvement of the discs and posterior ligaments associated with secondary deformity	IV
Dai et al. [8] (2001)	Operative and non-operative treatment of thoracic spine fractures	Retrospective case series	53 non-op vs. 24 op	24—180	“Unstable” fractures should be stabilized operatively	IV
Schouten et al. [36] (2014)	Quality of life after thoracic fractures (non-operative and operative treatment)	Cross-sectional cohort study based on prospective database	28 (non-op) vs. 98 (operative)	12–186	Patients with ASIA D or E recover fully in general health They have a favorable prognosis compared with other spinal injuries	III
Huckell et al. [20] (1994)	Distraction rod vs Luque rod fixation of thoracic spine fractures	Retrospective case series	34	> 6 months	At final follow-up kyphosis returned to the preoperative values in both groups	IV
Yue et al. [46] (2002)	Operative treatment of thoracic spine fractures (incl. L1)	Retrospective case series	32	22	Posterior pedicle screw fixation of thoracic fractures is safe	IV
Payer et al. [29] (2005)	Operative treatment of upper thoracic spine fractures	Retrospective case series	8	15	Posterior pedicle screw fixation of upper thoracic fractures is safe	IV-
Fisher et al. [13] (2009)	Operative treatment of upper thoracic spine fractures	Retrospective case series	27	28	Posterior pedicle screw fixation of upper thoracic fractures is safe end efficacious	IV
Marré et al. [28] (2011)	Operative treatment of thoracic spine fractures	Retrospective case series	51	12	Neurological impairment was the most important predictor of complications and disability	IV
Vassal et al. [41] (2014)	Operative treatment of thoracic spine fractures	Retrospective case series	50	23	Deformity can be seen during follow-up Restitution of the anterior column must be considered more frequently Quality of life at follow-up did not correlate with initial deformity	IV
Zhang et al. [47] (2015)	Timing of operative treatment of C3 thoracic fractures	Retrospective cohort study	36	23	No superiority of early (< 72 h) fixation in C3 thoracic fractures	IV
Gattozzi et al. [14] (2018)	Operative treatment of upper thoracic spine fractures	Retrospective case series	43	12	Surgical treatment of upper thoracic spine fractures is safe and effective	IV
Ghasemi et al. [15] (2016)	Operative treatment of thoracic spine fractures	Retrospective case series	25	15 (6–22)	Kyphotic deformity can be corrected by pedicle screw fixation Neurologic symptoms might recover	IV

### Biomechanics

A total of five studies dealt with mainly biomechanical aspects of posterior stabilization of thoracic fractures (Table 1). Generally, the thoracic spine is biomechanically stiffer than the other regions of the spine, because of two anatomical characteristics: the first one is the articulation of the head of the ribs with the articular facets of the adjacent vertebral bodies in combination with the radiate ligaments, which attach to the head of the ribs and both adjacent vertebral bodies, and the costotransverse ligaments. The second is the structure of the thoracic cage itself, which increases the resistance to all directions of motion [44]. Watkins et al. [43] evaluated the amount of stability provided by the rib cage and the sternum. In their study, the intact rib cage provided 40% of the stability of the thoracic spine in flexion–extension, 35% in lateral bending and 31% in axial rotation. A sternal fracture decreased the stability of the thoracic spine significantly by 42% in flexion–extension, 22% in lateral bending and 15% in axial rotation. Berg et al. [3] observed two clinical cases of combined sternal and thoracic spine fractures, which developed significant kyphotic deformities after nonoperative treatment and postulated the sternal-rib complex as the fourth column of the spine based on the three column theory of the spine by Denis. In general, the structural instability of thoracic fractures is treated with posterior instrumentation 2 levels above and below the fracture site, but in case of intact rib cage a short segment fixation with 1 level above and below the fractured vertebra could be an alternative. Therefore, Perry et al. [30] created a burst fracture at T9 in eight human thoracic spines (C7–L1) with intact rib cages and tested a long segment instrumentation (3 above, 2 below), a short segment instrumentation (1 above/1 below) with and without vertebral augmentation and vertebral augmentation without instrumentation. In their study, the long segment instrumentation showed a significant reduction of ROM during flexion–extension (− 90%), whereas the other instrumentations only tended to reduce motion. However, Perry et al. [30] suggested that in case of intact rib cages short segment instrumentation might adequately stabilize the spine. A common strategy to increase stability of short segment fixation is the addition of cross-links or screws at fracture site (index screws). Lazaro et al. [24] evaluated seven human thoracic spine segments after creating a wedge fracture in five conditions: long segment fixation (2 above/2 below) with cross-link, short segment fixation (1 above/1 below), short segment fixation with cross links, short segment fixation with index screws and short segment fixation with index screws and cross-link. The long segment fixation was significantly stiffer than short segment fixation, but adding index screws to the short segment construct significantly improved stability by 25%. Adding the cross-link increased stability only during axial rotation. Alternative fixation devices or techniques for the thoracic spine have been described, but pedicle screw systems still provide superior biomechanical properties [18, 26]. All relevant articles are summarized in Table 1.

### Placement of thoracic pedicle screws

#### Transpedicular pedicle screws

Seventeen studies evaluated screw positioning, screw implantation and intra-operative control of screw placement (Table 2). The Placement of pedicle screws in the straight ahead technique promoted by Roy-Camille et al. [34] is associated with a penetrating rate of 41% [40]. Generally, structures at risk were the intercostal vessel (T4–5), esophagus (T5–9), diaphragm, azygos vein (T5–11), inferior vena cava (T11–12) on the right side as well as aorta (T5–12) and esophagus (T4–9) on the left side.

Dwahan et al. [9] analyzed the effective pedicle diameter and the mean insertion angle comparing three types of insertion techniques (straight ahead, straight forward with angulation in the axial plane, and anatomic with angulation in the axial and sagittal planes) and found the largest effective diameter using the anatomic way.

Additionally, the funnel technique, opening the insertion point to visualize the medial cortex of the lamina, was analyzed in a cadaver study [6]. The authors had a low perforating rate of 10% grade 1 and 0.4% grade 2. Another way to improve the accuracy could be the use of a special drill guide as tool for thoracic pedicle screw placement [27]. Only 5 of 66 screws had a perforation, all less than 2 mm.

Lehmann et al. [25] analyzed 229 pedicles in an anatomic study. The authors found an ideal starting point 2–3 mm lateral to the midline of the superior articular facet (line between the lateral and the middle third of the superior facet). The cephalocaudal point is more level depending. T7–9 at the cranial border of the transverse process, T6 and T10 between the cranial border and proximal one-third of transverse process, T4–5 and T11 proximal one third of transverse process and T1–3 and T12 bisected transverse process.

In a clinical setting, Bransfold et al. [4] reported of 1.2% revision surgery rate due to screw misplacement in 245 patients treated because of thoracic fractures in an open technique under fluoroscopic control. Thereby, there seems to be a correlation between pedicle diameter and penetration rate with a 33% misplacement rate in pedicle diameters of less than 5 mm to 11% in diameters between 5 and 7 mm and no misplacement in diameters above 7 mm [16]. Altogether, there seems to be no difference in accuracy of screw placement in thoracic spine between the open and percutaneous technique [23].

Two studies investigated the benefit of navigation for pedicle screw placement in the thoracic spine [35, 45]. Both studies found significant higher accuracy in the navigated pedicle screw placement technique. Alternatively, intraoperative 3D-Imaging using a cone-beam device can be used to improve accuracy [2]. A total of 3.8% of the pedicle screws were re-implanted due to the findings in the 3D-Scan. No penetration of more than 2 mm was seen postoperatively. Additionally, Fischer et al. [12] used preoperative CT-guided transpedicular guide wires and reported a high accuracy and a low complication rate.

In contrast, intraoperative electromyographic monitoring could not improve the accuracy of transpedicular screw placement in thoracic spine [31].

#### Parapedicular pedicle screws

The insertion of pedicle screws parapedicularly is an alternative to the transpedicular screws placement [10]. The accuracy of this technique under computer-assisted navigation was good and reliable [22].

Husted et al. [21] performed a cadaver study for the parapedicular approach. In this technique, the screws were inserted cephalad to the tip of the transverse process and advanced between the transverse process and the rib. The direction of insertion was caudad in an oblique direction following the course of the rib medially to its articulation with the vertebral body under fluoroscopic control. All screws had an extraspinal position and were positioned in the pedicle rib unit.

#### Translaminar screws

Alternatively, translaminar screw fixation has been evaluated, which can be inserted with high accuracy under clinically control [7].

Hu et al. [19] analyzed the laminar of the high thoracic spine (Th 1–3) in an Asian population. The authors found larger lamina in males than females but sufficient corridors in all cases.

### Outcome after posterior stabilization

There were 14 clinical studies evaluating the outcome in patients with unstable thoracic fractures (Table 3). Most studies on non-operative treatment of unstable fractures are historical. They report treatment algorithms that include bedrest for 2–6 weeks [5, 17, 48]. Hospitalisation times with non-operative treatment range from 3 weeks to 3 months [5, 17] and can be reduced to between less than 2 weeks and 3 weeks by operative treatment in patients with isolated thoracic vertebral fractures [13, 14].

After surgical treatment of thoracic vertebral fractures by posterior stabilization in general, non-surgery-related complications occur in 49%–60%, especially in patients with complete or incomplete paraplegia [28, 46]. Deep venous thrombosis is observed in 4%–9% [13, 36]—compared to 24.5% in conservatively treated patients [5]. This results in an in-hospital mortality rate of 6%–8% [46, 47]. Neurologic worsening during follow-up can be reduced from around 2% [5, 48] with non-operative treatment to less than 1% with posterior stabilization [28, 29, 45, 46]. The only complication directly linked to the non-operative treatment is brace-related skin complications in 16% [5]. For surgical posterior stabilization, the need for revision surgery is reported in 0 to 22% [13, 15, 29, 36, 41, 46]. Some authors differentiate between early revision surgery in 4%–19% and late revision surgery in 16%–22%—with late revisions being mainly those for pain and low-grade-infection [13, 14, 28]. One study identified lamina hooks as source of potential neurological complications requiring revision surgery [41].

Radiological secondary deformity was seen in 94% with non-operative-management, particularly in fractures with associated injuries to the adjacent disc and/or posterior ligaments [17, 48]. With posterior stabilization, a loss of reduction of 1° to 4° was observed after 1 year and of 2° to 4° after 2 years and more [13, 14, 29, 41]. The only study reporting data on bone healing stated a fusion rate of 95% at 12 months on conventional radiographs [14].

Pain at follow-up after non-operative treatment was reported by 21% to 48% of the patients at 5 years and longer, while this was the case in 35% with a mean of VAS 3 at 15 months after operative treatment with posterior stabilization [5, 8, 17, 48]. Harkönen et al. [17] reported that 13% of the patients had “poor” mobility at 5 years with non-operative treatment, while Yue et al. [46] reported “very good to excellent levels of satisfaction with regards to pain, mobility, posture, and activity” 22 months after posterior stabilization.

Only few studies report patient-reported outcomes for operatively treated patients with values for the SF 36—PCS of 36–40 and the SF 36-MCS of 43–56 [36, 41]. No difference in functional outcome was seen between short and long segmental construct. Generally, return to work was less likely in patients with concomitant spinal cord injuries (25% of ASIA A/B/C and 88% of ASIA D/E) [36]. One year after posterior stabilization of a thoracic vertbral fracture, 7.8% received compensation payments for chronic back pain [28].

There is also some evidence that percutaneous posterior stabilization of thoracic spine fractures is associated with a reduced inflammatory response, less bleeding, shorter hospitalisation time and earlier return to activities of daily living [20, 42].

## Discussion

The majority of articles that were selected had a low level of evidence (level IV). Thus, no strength of evidence and statistical precision in the evaluation of the outcomes was performed. In contrast, a narrative presentation of the results was chosen.

Based on this, the most important findings of this study are the low evidence on posterior stabilization of mid-thoracic vertebral body fractures particularly dealing with clinical outcomes can be observed. Besides, the tremendous effect of sternal integrity on spinal stability has been shown. Furthermore, the penetration rates of pedicle screws varies between the studies ranging up to 40% based on the rather small pedicle diameters at the upper thoracic spine. Thereby, the accuracy of pedicle screw placement could be improved by navigated pedicle screw insertion as well as intraoperative 3D-Imaging and the insertion should be done anatomically.

Most authors perform long-segmental posterior instrumentation in patients with mid-thoracic vertebral fractures [4, 29, 38, 41]. This seems to be necessary particularly in patients with concomitant sternal fractures [30]. Based on the findings on the association of pedicle diameters and penetration rates, navigated techniques or intraoperative 3D-Imaging should be particularly used in pedicle diameters of less than 5 mm [2, 16, 35, 45]. In small pedicles, translaminar screws at the upper thoracic spine or parapedicular screws might be a viable alternative [7, 22]. Interestingly, percutaneous techniques were not associated with higher penetration rates. Therefore, percutaneous stabilization is a very good solution in long-segmental stabilizations and polytraumatized patients based on their reduced approach-related morbidity and significantly reduced blood loss [23].

Under consideration of these results, the authors generally recommend long-segmental stabilization of unstable midthoracic fractures in patients with concomitant fractures of the rib cage, particularly sternal fractures. On the other side, comparable biomechanical construct stabilities could be seen in unstable midthoracic fractures with intact rib cages between short-segmental stabilization with index screw and cross link and long-segmental stabilization. However, it has to be kept in mind that biomechanical tests are traditionally performed under ideal conditions, including optimal pedicle screw positioning. In contrast, it has been shown that pedicle screw placement was insufficient in a relevant number of patients with small pedicle including a penetration rate of 33% in pedicle diameters of less than 5 mm [16]. This can affect the screw hold considerably leading to impaired construct stability in patients treated with short-segment stabilization. Based on the limited pedicle screw diameters at the midthoracic spine, short-segmental stabilization has to be discussed critically in this region particularly as there are no signs of any functional benefit of short-segmental stabilization [36, 38]. In the authors’ view short-segment stabilization is only a viable option in patients with large pedicle diameters, or in cases where navigation or intraoperative 3D-scans are used. In these cases, correct pedicle screw placement can be expected. Based on the biomechanical data short-segment constructs should include index screws. Additionally, cross links improve the construct stability and should be added particularly when an open approach has been used. Furthermore, pedicle screws with a diameter as large as possible and a sufficient length should be used to increase construct stability. In the authors’ experience, no pedicle screws with diameters of less than 5 mm should be inserted. Thereby, the authors recommend parapedicular screw placement in those patients with too small pedicle diameters.

Unfortunately, the evidence level of the clinical follow-up studies is low. Altogether, the results of posterior stabilization of unstable thoracic fractures are superior compared to non-operative treatment, with a lower rate of neurologic deterioration, higher return to work rates and lower limitations [28, 29, 46, 47]. However, the complication rate after both, operative and non-operative treatment is high [26, 41, 47].

This study has several limitations. First of all, articles might have been missed by the used search items. Besides, the level of evidence in the majority of studies is low, leading to a limited conclusion that can be drawn out of it. Last but not least, the high number of studies with low evidence level was the reason to present the results in a narrative manner without any statistical evaluation of the strength of evidence and the precision of outcome parameters.

Altogether, further studies are necessary to define patients who benefit from surgery as well as which surgical strategies might lead to superior mid- and long-term results.

## Conclusions

The evidence of the available literature is low. Prospective randomized studies are lacking. However, long-segmental stabilization is indicated in unstable midthoracic fractures with concomitant sternal fractures. Generally, long-segmental constructs seem to be the safer treatment strategy considering the relative high penetration rate of pedicle screws in this region. In the case of short-segmental stabilization, the use of index screw can be recommended. Additionally, pedicle screws with a diameter as large as possible should be used. Thereby, navigated insertion techniques and intraoperative 3D-imaging help to improve pedicle screw placement accuracy.
